# NKp44-Derived Peptide Used in Combination Stimulates Antineoplastic Efficacy of Targeted Therapeutic Drugs

**DOI:** 10.3390/ijms232214054

**Published:** 2022-11-14

**Authors:** Muhammed Iraqi, Priyanka Bolel, Rhitajit Sarkar, Baisali Bhattacharya, Muhammad Abu Ahmad, Avishay Edri, Laila C. Roisman, Moshe Elkabets, Walid Shalata, Nir Peled, Angel Porgador

**Affiliations:** 1 The Shraga Segal Department of Microbiology, Immunology, and Genetics, Faculty of Health Science, Ben-Gurion University of the Negev, Beer Sheva 84105, Israel; 2 National Institute for Biotechnology in the Negev, Ben-Gurion University of the Negev, Beer Sheva 84105, Israel; 3 National Institute of Diabetes and Digestive and Kidney Diseases, National Institutes of Health, Bethesda, MD 20892, USA; 4 Shaare Zedek Medical Center, Oncology Division and Cancer Institute, Jerusalem 9103102, Israel; 5 The Legacy Heritage Center & Larry Norton Institute, Soroka Medical Center, Beer Sheva 84105, Israel

**Keywords:** lung cancer, NKp44, PCNA-binding peptide, personalized medicine, synergistic effect, tumor xenograft

## Abstract

Lung cancer cells in the tumor microenvironment facilitate immune evasion that leads to failure of conventional chemotherapies, despite provisionally decided on the genetic diagnosis of patients in a clinical setup. The current study follows three lung cancer patients who underwent “personalized” chemotherapeutic intervention. Patient-derived xenografts (PDXs) were subjected to tumor microarray and treatment screening with chemotherapies, either individually or in combination with the peptide R11-NLS-pep8; this peptide targets both membrane-associated and nuclear PCNA. Ex vivo, employing PDX-derived explants, it was found that combination with R11-NLS-pep8 stimulated antineoplastic effect of chemotherapies that were, although predicted based on the patient’s genetic mutation, inactive on their own. Furthermore, treatment in vivo of PDX-bearing mice showed an exactly similar trend in the result, corroborating the finding to be translated into clinical setup.

## 1. Introduction

Non-small-cell lung cancer (NSCLC) is the primary (~85%) histological subtype of lung adenocarcinoma in both men and women, causing more than 1.5 million deaths worldwide [1]. NSCLC has two most prominent subtypes—lung adenocarcinoma (LUAD) and lung squamous cell carcinoma (LUSC) [2]. In earlier stage of the disease, cytotoxic regimens have demonstrated their greatest effect. Segmentectomy is the most effective therapy for stages I to II and selected cases of stage IIIA NSCLC [3]. Adjuvant cytotoxic therapy with a cisplatin-based combination doublet has been the standard therapy for patients with advanced stage NSCLC, as well as being used as maintenance therapy in patients with non-LUSC histology who achieve tumor control after the initial four to six cycles [4]. The standard therapy for patients with unresectable locally-advanced NSCLC is the combination of cytotoxic therapy and thoracic radiation [5,6]. However, despite its initial curative progression, a high percentage of tumors will recur, with 5-year overall survival ranging from 83% for stage IA to 36% for stage IIIA disease [7]. While generalized medicine with traditional chemotherapy yielded comparatively poor response rates and treatment results, the identification of targetable gene alterations has transformed the management of lung cancer, with the incorporation of tumor genotyping to allow personalized/individualized therapy leading to remarkable responses. In the multicenter Lung Cancer Mutation Consortium, targetable oncogenic drivers were observed in 64% of patients with LUAD, for whom the use of genotype-directed therapy was associated with improved survival compared to those treated without targeted therapies [8].

Proliferating cell nuclear antigen (PCNA) is the eukaryotic sliding clamp [9,10] that plays an essential role in different cellular processes, such as chromosomal DNA replication, DNA repair, cell cycle control, apoptosis, chromatin metabolism, and gene expression [9,11,12]. PCNA expression is upregulated in cancer cells compared to healthy normal cells and can be used as a target for the development of anti-proliferation and anti-cancer drugs [13,14,15,16,17,18,19,20,21,22]. Wang et al. [23] showed that PCNA is highly expressed in NSCLC, via STAT3-activation-promoted tumorigenesis, thus making PCNA a prognostic marker for NSCLC as well as a molecular target. Hence, targeting PCNA using synthesized peptide molecules has led to a more profound impact on arresting cancer cell growth [17,24]. These peptides are derived either from functional binding domains within PCNA or from conserved binding motifs, found within the protein ligands of PCNA [17].

PCNA can be recognized by NKp44 protein (natural cytotoxicity triggering receptor 2; NCR2), leading to inhibition of natural killer (NK) cell’s activity against cancer cells [25]. In our previous study, we developed a cell-penetrating NKp44-derived linear peptide also capable of cell penetration, R11-NLS-pep8, which can specifically interact with nuclear PCNA and mediates tumor cell death [26]. In the literature, it was shown that peptides targeting nuclear PCNA involved in DNA repair can sensitize cancer cells to existing anti-cancer therapeutics [27,28]. Thinking on the same line, in this study, we found that our designed PCNA-targeting peptide, R11-NLS-pep8, has increased the efficacy of therapeutic drugs for lung cancer treatment by the synergistic killing of tumor cells. To test the synergistic effect of the indigenously developed peptide in combination with therapeutic drugs in PDX-derived explant samples, we have employed a highly efficient drug screening method of Tumor Ex Vivo Analysis (TEVA) which was developed by the reporting laboratory [29]. Our current study shows that a PCNA-binding peptide in a combinatorial mode exerts enhanced efficacy of the targeted therapy regime for any individual NSCLC patient, in an accurate and timely fashion.

## 2. Results

### 2.1. Combination of Peptide with Chemotherapies Produced Variable Results Ex Vivo

**Figure 1:**
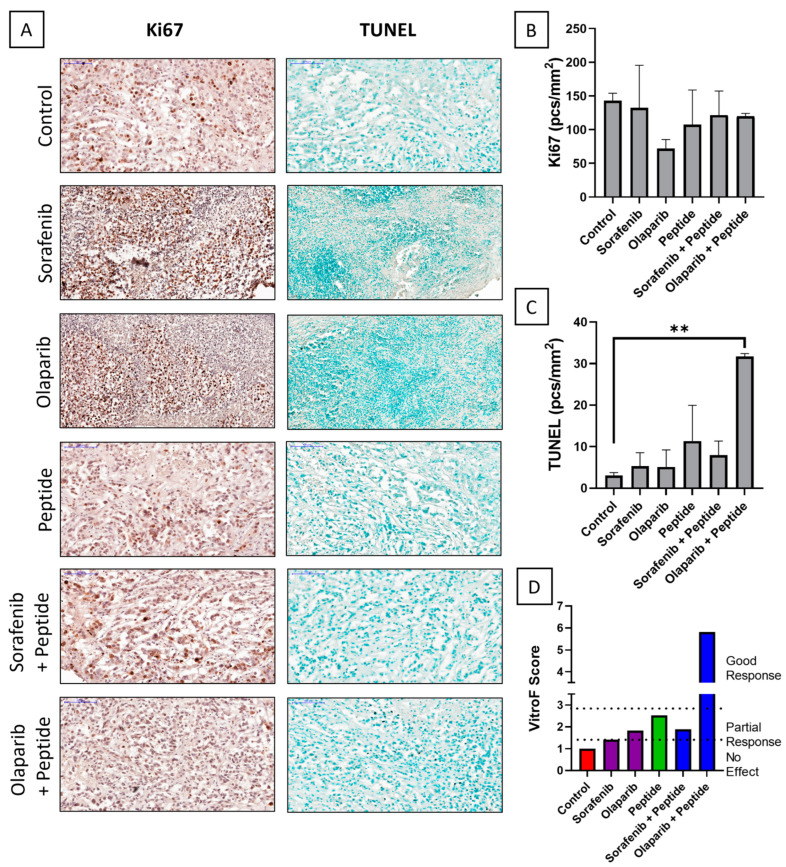
IHC staining of tissue explants of Patient #1 after ex vivo single and combinatorial drug treatment with olaparib, sorafenib and R11-NLS-Pep8. (**A**) Representative IHC images of control and treated explants showing cell proliferation marker, Ki67 and TUNEL assay (20× magnification, 100 μm scale-bar). Quantification of (**B**) Ki67 and (**C**) TUNEL positive cells of control and treated explants, expressed as object frequency (pcs/mm^2^). Graphs representing mean ± SEM. ** *p* < 0.01, with ordinary one-way ANOVA multiple comparisons test, is significantly different from Control. (**D**) VitroF score of the treatments.

**Figure 2:**
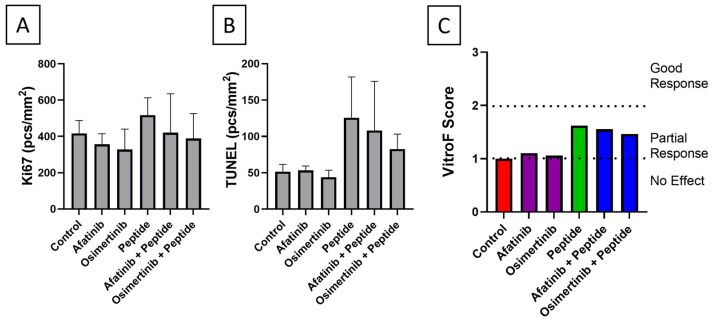
Quantification of ex vivo response of tissue explants of Patient #2 for single and combinatorial treatment of afatinib, osimertinib and R11-NLS-Pep8. Graphs (mean ± SEM) showing quantification of (**A**) Ki67 and (**B**) TUNEL positive cells of control and treated explants, expressed as object frequency (pcs/mm^2^). (**C**) VitroF score of the treatments.

**Figure 3:**
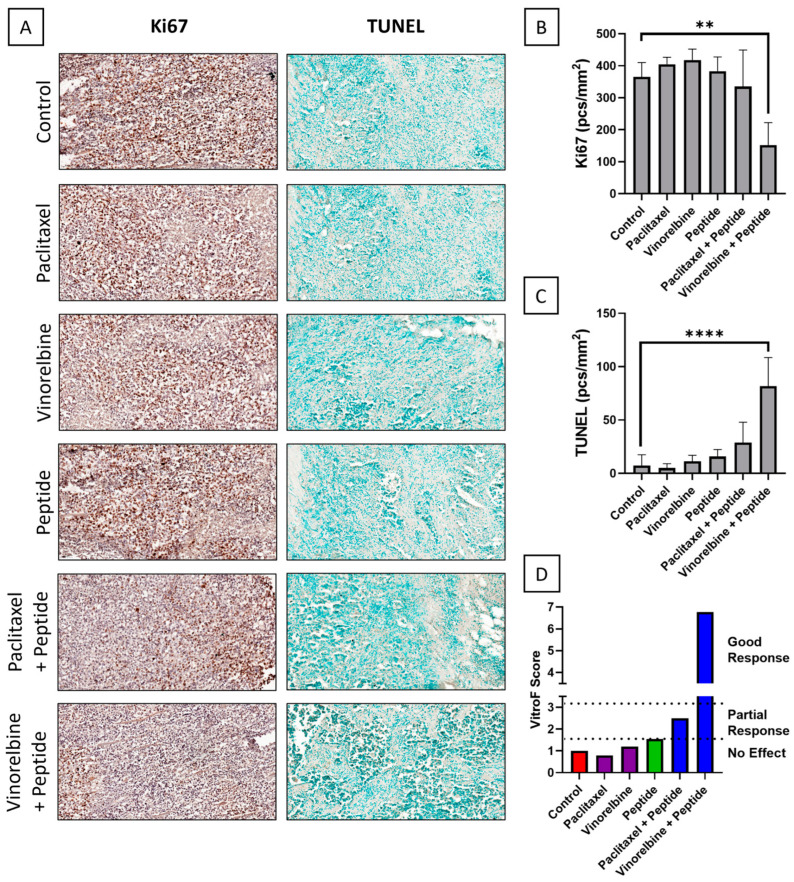
IHC staining of tissue explants of Patient #3 after ex vivo single and combinatorial drug treatment with paclitaxel, vinorelbine and R11-NLS-Pep8. (**A**) Representative images of control and treated explants showing cell proliferation marker, Ki67 and TUNEL assay (20× magnification, 100 μm scale-bar). Quantification of (**B**) Ki67 and (**C**) TUNEL positive cells of control and treated explants, expressed as object frequency (pcs/mm^2^). Graphs representing mean ± SEM. ** *p* < 0.01 & **** *p* < 0.0001 with ordinary one-way ANOVA multiple comparisons test, is significantly different from Control. (**D**) VitroF score of the treatments.

In order to investigate the synergistic killing effect of the combination of therapeutic agents and the peptide R11-NLS-pep8, ex vivo experiments were performed on PDXs from the patients using TEVA model. There were variable responses on the samples treated with or without the peptide for available chemotherapies predicted on the basis of genomic analysis for each patient. They are as follows:

- Patient #1
- Given the patient’s advanced age and possible adverse effect due to platinum sensitivity, only immunotherapy was performed on the patient owing to possible adverse side effects of existing chemotherapy. Keeping in mind the KRAS G12V mutation in the patient, the standard KRAS downstream inhibitors sorafenib and olaparib were determined as the second line of treatment individually or in combination with R11-NLS-pep8. In the IHC images of the TEVA model (Figure 1A), we find that although there is no decrease in Ki67 activity, there is substantial cell death via TUNEL activity in the treatment of 5 µM olaparib with 4 µM R11-NLS-pep8. The quantification of Ki67 (Figure 1B) and TUNEL (Figure 1C), in terms of object frequency (pcs/mm^2^), corroborated to the fact that neither sorafenib nor olaparib exerted the cell killing that was achieved by olaparib in combination with the peptide. The VitroF score, obtained by combining the scores from Ki67 and TUNEL results, suggested that only the combination of olaparib with the peptide presented good response.

- Patient #2
- After failure with antimetabolites (pemetrexed) and DNA synthesis inhibitor (carboplatin) in the first line of treatment, the patient was treated with erlotinib and osimertinib based on the epidermal growth factor receptor (EGFR) T790M mutation reaching limited success. Throughout the treatment regime, pembrolizumab was employed to compensate for the tumor’s ability to immune evade due to high PD-L1 load. In our study, we focused on the primary EGFR mutation and used a broad-spectrum EGFR blocker, afatinib, and previously used Osimertinib which showed little success in the patient’s treatment regime. The results showed little difference between the used drugs and their combination with the peptide in terms of Ki67 and TUNEL staining (Figure 2A,B). Consequently, the VitroF score (Figure 2C) predicted a partial response of the drugs in combination with the peptide in comparison to their stand-alone use.

- Patient #3
- Along with the more aggressive LUSC type of NSCLC, this patient had KRAS, STK11, and CDC73 type mutations. The first line of treatment with more potent DNA synthesis inhibitors, carboplatin, paclitaxel, and gemcitabine failed, despite combination with pembrolizumab to counter high PD-L1 load. Further immunotherapies with combination of immune checkpoint blockers, viz. nivolumab and ipilimumab still produced unpromising results. In our study, we used paclitaxel, a widely used microtubule binder that inhibits DNA synthesis, and vinorelbine, another alkaloid microtubule toxin. The immunohistochemical images from TEVA showed (Figure 3A) that only the combination of 100 µM vinorelbine with R11-NLS-pep8 (4 µM) lessened the extent of Ki67 staining and increased apoptotic cells. This was not observed in any of the standalone alkaloids at the same concentration and also for the same class of compound (paclitaxel) in combination with the peptide. The object frequency for Ki67 (Figure 3B) and TUNEL (Figure 3C) followed the same pattern as that of the IHC images (*n* = 3). The subsequent VitroF score (Figure 3D) observed a good response for the combination of vinorelbine with R11-NLS-pep8 over all other treatments used for this patient’s TEVA.

### 2.2. Patient Tumors Show Variable Degree of PD-L1 and Surface PCNA Expression

**Figure 4:**
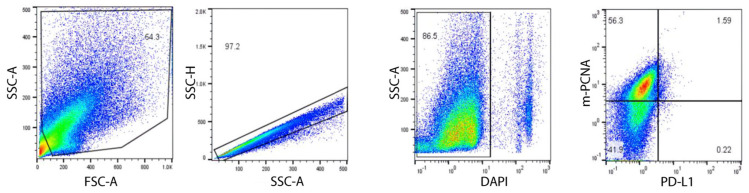
Flow cytometry scheme and representative dotplots (Patient #1) for expression of membranal PD-L1 and PCNA (m-PCNA) on digested tumor cells from PDX samples on cell surface.

When subjected to flow cytometric analysis, the cells derived from the patients’ tumors showed differential staining for all the patients (Figure 4). Only live cells (DAPI negative) were used in the analysis of PD-L1 vs. membrane-associated PCNA using mAb14 which is previously reported to specifically stain membrane PCNA [30].

The summary of the staining for each patient, as showed in Table 1, demonstrated that both Patients #1 and #3 had high m-PCNA expressed on cell surface, whereas only the latter had high PD-L1 expression. Neither expression was found highly evident for the cells derived from patient #2.

### 2.3. Findings from TEVA Are Demonstrated in Immunocompromised Mice Model

**Figure 5:**
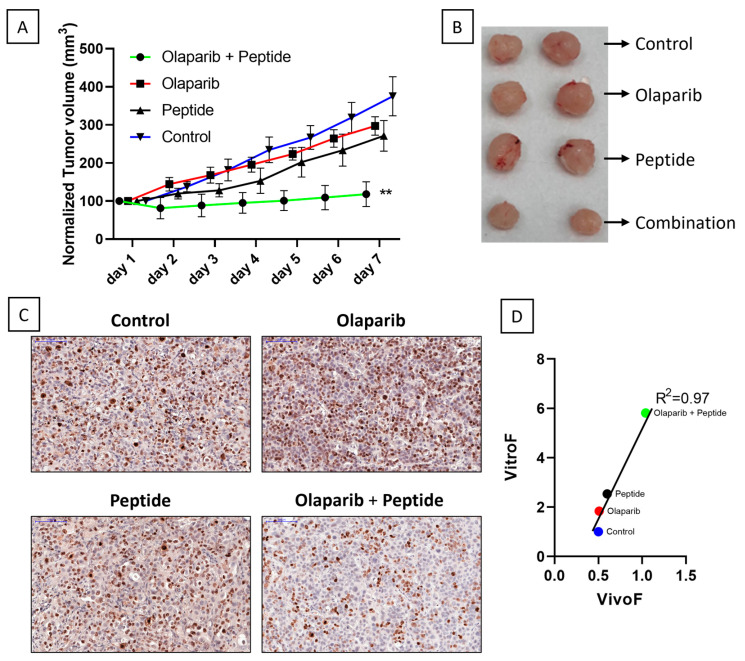
Correlation between in vivo and ex vivo response of Patient #1 to single and combinatorial treatment of therapeutic drug and R11-NLS-Pep8. Xenografts bearing NSG mice were treated with Olaparib, R11-NLS-Pep8, and a combination of both for 7 days. Olaparib was administered daily while R11-NLS-pep8 was administrated thrice in a week. Both the materials were injected intraperitoneally (IP). (**A**) Mean tumor volumes (mm^3^) normalized to day 1 measurement of each mouse in Control and drug-treated groups are shown in the tumor growth curve. Graphs representing ordinary one-way ANOVA multiple comparisons test, ** *p* < 0.01, significantly different from control. (**B**) Comparison of the tumor sizes from one representative mouse in each group after final excision. (**C**) Representative IHC images of control and treated explants showing cell proliferation marker, Ki67 post in vivo treatment (20× magnification, 100 μm scale-bar). (**D**) Linear regression plot for the correlation data between VitroF and VivoF for each treatment in Patient #1 (GraphPad 8.0). The statistical significance of the correlation was determined using the correlation coefficient (R^2^ = 0.97).

For establishing the practical use of R11-NLS-pep8 in vivo, we transplanted NSG mice with PDX from Patient #1 and treated mice with previously reported doses of the chemotherapy drug and combination used in the TEVA model [26,31]. The PDX-bearing mice were allocated into four groups and treated with vehicle (received only appropriate solvent), olaparib and R11-NLS-pep8 as single and combination treatments for 7 days. In Figure 5, it can be observed that there is gradual and significant decrease of the tumor volume in the mice receiving combinatorial treatment of 20 mg/kg body weight olaparib and 5 mg/kg body weight R11-NLS-pep8 compared to the mice receiving single-agent treatment of olaparib and R11-NLS-pep8 or mice in Control group (Figure 5A). At the end of the study, tumors were excised and photographed (Figure 5B) which clearly showed significant difference in the tumor sizes in the combinatorial drugs-treated group compared to single treatment or the Control group. The extent of proliferation in the tumor tissues as indicated by Ki67 assay (Figure 5C) was also in agreement with these results.

A correlation (Figure 5D) of the VivoF and VitroF scores was completed to compare the outcomes of in vivo treatment to those of the ex vivo assay, respectively. Fundamentally, the first and last measurements for each PDX were normalized to the average of vehicle-treated group and formulated together to give the VivoF. Combinatorial treatment of olaparib and R11-NLS-pep8 was the most potent treatment in both the ex vivo and in vivo assays as can be found from the regression curve showing a significant correlation between VitroF and VivoF (R^2^ = 0.97).

## 3. Discussion

Proliferating cell nuclear antigen (PCNA) regulates oncogenesis [23] by acting as a central coordinator of DNA replication, repair, chromatin dynamics, and cell cycle regulation [32,33]. PCNA expression is associated with poor 5-year survival, higher WHO grade [34], and is correlated with tumor stage, differentiation degree, pathological type, and metastasis in a clinical setup [35]. Given that it is already established as a useful prognostic and diagnostic biomarker or an effective therapeutic target in various cancers, substantial efforts have been made to develop novel approaches targeting PCNA [36] for cancer therapy. Therefore, a plethora of peptides mimicking the APIM or a sequence of caPCNA (“cancer-associated PCNA”) [28,37,38,39,40], targeting the PIP-box or caPCNA [41,42,43,44] were generated that selectively inhibit tumor cell growth, induce apoptosis, and enhance cytotoxicity of chemotherapy drugs on tumor cells. They are also well-tolerated during animal treatment, especially when combined with DNA damage drugs [27,28,40,43]. We have already shown that R11-NLS-pep8 can reduce cell viability and promote cell death in various murine and human cancer cell lines and inhibit tumor growth by interfering with the function of intracellular PCNA in the 4T1 breast cancer and the B16 melanoma in vivo models. It is also to be noted that R11-NLS-pep8 originated from an extracellular immune protein and does not contain or target the PIP or APIM motifs [26,45,46].

Our study was conceived with the notion that patients with advanced NSCLC seldom respond to the therapies (chemo- or immuno-) selected based on their existing oncogenic mutations, i.e., targeted therapies. For the two patients that were responsive in our models, the most prevalent was *KRAS* mutation. KRAS mutations occur in 31% of unresected treatment-naïve lung adenocarcinomas [47,48] and involve multiple cellular pathways. KRAS mutations are dominant in lung, colorectal, and pancreatic cancers [49] with various types of mutations. G12V mutation [50], carried by Patient #1 in our study, represents around 22% of all KRAS mutations involving the replacement of glycine by valine. In the less common G12D mutation (16%) found [51] in Patient #3, a glycine is replaced by an aspartic acid mutation resulting from a G>A substitution. Although KRAS inhibitor therapies exist [52,53,54], they have faced challenges due to the difficulty of targeting RAS directly and whether the KRAS mutant cancers retain dependence on KRAS, thus giving rise to a need for combinatorial strategies. Contradictory evidence exists to show inhibition of KRAS leading to tumor regression [55,56,57,58], and KRAS-mutant cell lines exist whose growth and tumorigenicity do not depend on oncogenic KRAS [59,60,61], on the other hand. Studies pose the question that the initiation of oncogenic transformation and maintenance of the transformed state is separable and that KRAS dependency is not a fundamental trait of KRAS-induced tumors [62,63,64,65]. Hence, for Patient #1, although sorafenib and olaparib were chosen chemotherapies due to their advantages in advanced platinum-sensitive KRAS-mutant NSCLC [66,67,68,69] for our TEVA model, their standalone usage bore no significant results. On the other hand, KRAS^G12V^ mutation could induce PD-L1 expression and promote immune escape via transforming growth factor-β/EMT signaling pathway in KRAS-mutant NSCLC [70,71], indicating the failure of PD-L1 targeting therapy using Pembrolizumab. Moreover, our flow cytometry analysis shows that the cells isolated from Patient #1 are not PD-L1 rich, resulting in passive anti-PD-L1 immunotherapy. Now, recent studies have demonstrated several molecules that directly interact with KRAS regulate PCNA expression in lung, colorectal, and pancreatic cancers [72,73,74], and Caiola et al. [75] have shown that base-excision repair (BER) is involved in KRAS-mutated NSCLC, which results in PCNA ubiquitylation leading to the polymerase switch between replicative and translesion synthesis polymerases, initiating another important process contributing to crosslink repair [76]. So, we might rightfully assume that R11-NLS-pep8 interacts with PCNA to stop ubiquitylation and, in turn, translesion synthesis allowing olaparib to inhibit the poly (ADP-ribose) polymerase (PARP) resulting in DNA damage as found in the TUNEL studies. On the other hand, we find sorafenib, a TKI, not to act by inhibiting cell proliferation as reflected in an insignificant change in proliferation marker, Ki67.

Before the advent of immunotherapy, platinum-based doublet chemotherapy regimens were the standard first-line treatment option for metastatic NSCLC [77,78]. Then, standalone immune checkpoint inhibitors targeting programmed cell death-1 (PD-1) or its ligand (PD-L1), or in combination with chemotherapy, have transformed the treatment landscape for patients with metastatic NSCLC, especially those without oncogenic driver mutations [79,80,81,82,83,84]. However, immunotherapies still do not work for a significant patient population, primarily due to gene mutations and co-mutation patterns in individual patient response linked to standard chemotherapy and/or immunotherapy in advanced NSCLC [85,86,87,88,89,90,91,92,93]. In general, the serine-threonine kinase 11 (STK11, alias LKB1) mutation is present in about 10% of KRAS-driven NSCLC, more commonly in patients with a smoking history [94]. In NSCLC, mutations in STK11, followed by disruption of AMPK signaling pathways, are frequently observed [95,96]. Although STK11 is suggested to have roles in DNA repair pathways, the full extent of the influence of STK11 downregulation on DNA repair [97], pathways in lung cancer have not yet been explored. *STK11* mutations are often associated with an “immune-cold” tumor microenvironment with low PD-L1 and T-cell densities, high granulocyte colony-stimulating factor and IL-8 family cytokines, and production of neutrophil-like cells and myeloid cell-recruiting chemokines such as IL-6 [98,99]. Taken together, these effects account for the reason why the first line of platinum-based therapies, DNA-synthesis inhibitors, and extensive immunotherapies, including checkpoint-blockers, did not work for Patient# 3, although we see a high PD-L1 expressed in the cells derived from the patient’s tumor. Additionally, mutations of the Cell Division Cycle 73 (CDC73) [100] tumor-suppressor gene (previously known as HRPT2) are associated with the Hyperparathyroidism-Jaw Tumor (HPT-JT) syndrome, an autosomal dominant disease whose clinical manifestations are mainly parathyroid tumors and, less frequently, ossifying fibromas of the jaw, as well as uterine and renal tumors. It is seldom associated with NSCLC, esp. KRAS-driven and only bioinformatic analysis of the TCGA database shows that *CDC73* mRNA [101] expression was positively correlated with distant metastasis and unfavorable prognosis of lung cancer. Hence, the presence of this mutation in Patient #3 presumably did not contribute much other than feeble cell cycle regulation. Now, the literature shows [96,102] that the reliance of tumor cells on BER pathways comprising PCNA makes an attractive target for cancer therapy. Recently, Hurst et al. [103] showed that, on DNA damage, PCNA enhances microtubule polymerization via long-patch BER that requires longer strand synthesis by DNA polymerases, resulting in the perturbation of tubulin filament networks. So, we can safely predict that R11-NLS-pep8, when it binds to highly available cellular PCNA (as found by flow cytometry analysis), allows the tubulin network to be accessible by vinorelbine. The inability of BER due to PCNA-peptide binding resulted in arrested cellular synthesis as is reflected by reduced Ki67 staining, and the effect of vinorelbine to stimulate microtubule depolymerization [104,105] induced apoptosis of cancer cells as found from the TUNEL results in the TEVA analysis for this patient.

Considerable evidence [106,107] exists on targeting EGFR, a tyrosine kinase (TK) family protein, for the management of mutation-mediated NSCLC. Three generations of EGFR TK inhibitors have been developed to target EGFR mutations [108] to the kinase domain in NSCLC, although an ever-increasing number of mutation-mediated resistances are inevitable [109]. We find EGFR mutations regulate PCNA expression in NSCLC [110]; however, there is very little evidence of how PCNA is involved in TKI failure in EGFR-mutated cancers. By flow cytometry, we did not observe membrane-associated PCNA expression in the cells derived from patient #2’s tumor; this is associated with lack of binding to the tumor that is probably imperative to mediate peptide penetration into the tumor cell. In accordance R11-NLS-pep8 was non-functional to initiate any significant response using a variety of chemotherapy or immunotherapy.

Our study showed the synergistic killing effect of the combination of therapeutic agents and the peptide R11-NLS-pep8 using the TEVA model, a 3D tumor tissue explant culture, based on 24 h ex vivo drug exposure/treatment of PDXs allowing to test various single drugs and combinations robustly, and predict multiple drug responses accurately, as compared to in vivo treatment. In our study, PDXs from all the patients were not studied in vivo to correlate the ex vivo findings. This limitation should be considered to translate the finding in a more tangible way. Moreover, our proposal of pathways possibly activated by R11-NLS-pep8 is speculative based on the literature only. Nonetheless, we intend to conduct larger studies to address these current limitations and conclude that our study can be represented as a proof-of-principle that PCNA-binding peptide may make the already existing targeted therapy “better” when used in combination.

## 4. Materials and Methods

### 4.1. Patients and Tumor Samples Procurement

All three patients were diagnosed with stage IV lung cancer out of which two patients had adenocarcinoma and one patient had squamous cell carcinoma (Table 2). The study was conducted according to the guidelines of the Declaration of Helsinki and approved by the Institutional Review Board of Soroka medical center as off-label systemic therapy (Helsinki code of ethics 0384-18 and 0005-19 approved on 13 April 2021). Immediately after the surgery, solid tumor tissue samples were procured with patient consent and processed within 2 h of harvesting.

### 4.2. Mouse Strain and Establishment of Patient-Derived Xenografts (PDXs)

Sex unbiased six- to eight-week old immunodeficient NSG™ (NOD.Cg-*Prkdc^scid^ Il2rg^tm1Wjl^*/SzJ) mice were purchased from Envigo/Harlan Laboratories (Rehovot, Israel) and used for the study. Maintenance and breeding of all mice used in this study were carried out in the local animal care facility, approved by the Institutional Animal Care and Use Committee of Ben-Gurion University of the Negev, Israel.

The freshly harvested solid tumor tissue samples from patients were implanted subcutaneously in dorsal flanks of NSG mice to obtain the patient-derived xenografts (PDXs). A measurable size of tumor for the samples varied from 1 to 6 months. The tumors from mice of first generation were implanted in subsequent generations to maintain all the PDXs. All animal experiments were performed in accordance with the animal use guidelines and protocols approved by Institutional Animal Care and Use Committee (IACUC) of Ben-Gurion University of the Negev (BGU) aiming to ensure animal welfare and reduce suffering. The Animal ethical clearance protocol number used for this work is IL-80-12-2015.

### 4.3. Ex Vivo Tissue Explant Preparation and Drug Treatment

Tumor ex vivo analysis (TEVA) was performed when the volume of PDXs (preferably first generation PDX) reached ~500 mm^3^. The PDXs were excised aseptically from mice and cut into 2 mm × 2 mm × 2 mm tissue explants. The 2 mm × 2 mm × 2 mm explants were then incubated with different therapeutic drugs for 24 h in 48-well tissue culture plates at 37 °C, 95% relative humidity, and 5% CO_2_ in a CO_2_ incubator at sterile conditions with suitable control [29]. The 2 mm × 2 mm × 2 mm explants incubated only in culture media without any drug served as control. DMEM (Gibco, Billings, MT, USA) containing 20% FBS (Gibco), 1 mM sodium pyruvate (Biological Industries, Kibbutz Beit-Haemek, Israel), 2 mM L-glutamine (Biological Industries), 1% penicillin/streptomycin/amphotericin (Biological Industries), 0.1 mM MEM non-essential amino acids (Biological Industries), 10 mM HEPES (Biological Industries), BIOMYC-1 antibiotic solution (Biological Industries) and 50 µg/mL gentamycin (Gibco) was used as culture medium.

R11-NLS-pep8 (4 µM), sorafenib (25 µM, Bayer, Leverkusen, Germany), olaparib (5 µM, AstraZeneca, Cambridge, UK), afatinib (2 µM, Boehringer Ingelheim Pharmaceuticals, Amman, Jordan), osimertinib (2.5 µM, AstraZeneca, Cambridge, UK), vinorelbine (100 µM, K.S. Pharma, Erbil, Iraq), and paclitaxel (100 µM, Bristol-Myers Squibb, New York, NY, USA) were used in this study as therapeutic agents.

### 4.4. Tissue Microarray (TMA)

After 24 h incubation, the treated and untreated 2 mm × 2 mm × 2 mm tumor tissue explants were fixed in 4% paraformaldehyde and embedded in paraffin (FFPE) using automated tissue-processing machine (Leica Biosystems, Nußloch, Germany). TMA blocks containing 24 tissues were made from donor paraffin tissue blocks using 3 mm T-Sue^TM^ punch needles (Simport, Beloeil, QC, Canada).

### 4.5. Histological Analyses: Immunohistochemistry Staining and Quantification

Tissue sections of 5 μm thickness were cut from TMA blocks using fully automated rotary microtome (Leica RM2255). For Immunohistochemistry (IHC) staining, tissue sections were first deparaffinized by two rinses in xylene and 100% ethanol for 10 min each. After subsequent rinses in 70% and 50% ethanol for 5 min each, tissues were washed in ultrapure water and subjected to heat-mediated antigen retrieval at 95 °C for 30 min using antigen unmasking solution, citrate buffer pH 6.0 (Invitrogen, Waltham, MA, USA). Endogenous peroxidase activity was blocked with 3% hydrogen peroxide. Sections were then washed and ImmPRESS universal reagent (Vector Laboratories (Burlingame, CA, USA), Cat# MP-7500) was used according to manufacturer’s protocol for the blocking. After the blocking, sections were incubated with primary antibodies against Ki67 (1:200, Sigma Aldrich (St. Louis, MO, USA), Cat# SAB5500134). HRP conjugated secondary antibody from ImmPRESS universal reagent was used according to the manufacturer’s protocol. Lastly, staining was visualized by using DAB (DAB substrate kit, Cell Marque (Rocklin, CA, USA), Cat# 957D-60) according to manufacturer protocol, counterstained with hematoxylin and tissue sections were mounted with non-aqueous mounting medium (Vectamount, Cat# H-5000). The extent of tumor cell apoptosis was also studied by TUNEL assay according to manufacturer protocol (TREVIGEN (Gaithersburg, MD, USA), Cat# 4815-30-K).

TMA images were scanned by panoramic scanner (3D Histech, Budapest, Hungary) and analyzed by HistoQuant^TM^ software (3D Histech). For Ki67 and TUNEL staining, the software calculated the number of positive nuclei and the annotated area for each tissue and the value was expressed as object frequency (pcs/mm^2^).

### 4.6. Flow Cytometry

Analysis of cell surface PCNA and Programmed death-ligand 1 (PD-L1) expression was performed by flow cytometry. Three different NSCLC PDXs were harvested from NSG mice. After single cell separation by fine enzymatic and mechanical degradation, cells were washed twice with PBSX1 and passed through cell strainer to obtain a single-cell suspension mixture. Then, cells were co-stained with mAb14 [30] and anti-PD-L1 antibody. (Thermofisher (Waltham, MA, USA), Catalog # 14-5983-82, clone: MIH1) DAPI was used to distinguish live and dead cells. Flow cytometry was performed with FACSCanto II (BD Biosciences, Franklin Lakes, NJ, USA), and results were analyzed using FlowJo^®^ v.10.

### 4.7. In Vivo Evaluation of Drug Efficacy

Patient-derived xenografts were re-implanted subcutaneously into two dorsal flanks of 6–8 weeks old male NSG mice. When the tumor volume had reached ~200 mm^3^ mice were randomly divided into four groups with each group containing three mice keeping two restrictions; average PDX size and SD in the different groups should be similar, and SD should be kept lower, thus excluding from the experiment, prior to its beginning, mice bearing PDX with volumes at the edges of the PDX volume range. Group 1 was treated with olaparib (20 mg/kg, daily, intraperitoneally) and R11-NLS pep 8 (5 mg/kg, thrice weekly, intraperitoneally); group 2 was treated with only olaparib (20 mg/kg, daily, intraperitoneally); group 3 received PBS 1X with 10% DMSO (daily, intraperitoneally) and treated with only R11-NLS pep 8 (5 mg/kg, thrice a week, intraperitoneally); group 4 mice received PBS 1X with 10% DMSO (daily, intraperitoneally) served as the control. Treatment was continued for 7 days. Tumor sizes were measured daily using digital Vernier calipers in two perpendicular axes and reported as tumor volume, $V=\left(L\times W\times W\right)\times \left(\pi /6\right)$ in which L= longest axis, W= shortest axis. At the end of the study (i.e., day 7), tumor tissues were excised and processed for histological analysis.

### 4.8. Ex Vivo and In Vivo Scoring

The TEVA score, Vitro F, was calculated based on the staining of Ki67 and TUNEL of control (Ct) and treated (Tr) tissues,

TEVA Score Vitro F=0.5×CtKi67TrKi67+0.5×TrTUNELCtTUNEL

Ct (Control) = 100, Tr (Treatment) = Normalized value with respect to control.

Calculation of in vivo score, Vivo F, was based on the tumor growth rate on day 1 and end day (day 7) of the experiment: Control Tumor Volume (*CTV*) and Treatment Tumor Volume (*TTV*).

In vivo Score Vivo F=0.5×CTVTTVDay 1 measurement+0.5×CTVTTVDay 7 measurement

*CTV* (Control Tumor Volume) = 100, *TTV* (Treatment Tumor Volume) = Normalized value with respect to control.

### 4.9. Statistical Analysis

Graphical and statistical analysis were performed using GraphPad Prism 8.0 software. Graphs are represented as mean ± SEM. Statistical analysis of the data was performed using one-way ANOVA multiple comparisons test to determine the level of significance.

## Figures and Tables

**Table 1:** Extent of PD-L1 and m-PCNA staining in cells from all three patients.

Patient #	PD-L1%	m-PCNA%
1	2	56
2	4	7
3	86	30

**Table 2:** Summary of Clinical History and Treatment Plan of Patients Presented.

Patient #	Sex	Age	Smoking History	Histopathologic Diagnosis	Stage at Diagnosis	Genetic Alterations	Treatment		
							Chemotherapy	Immuno-Therapy	Targeted Therapy
1	Male	80	70 PY	Lung Adenocarcinoma	4-B	PD-L1 > 50%, KRAS G12V	No	Yes	No
2	Female	57	Never	Lung Adenocarcinoma	4-C	PD-L1 = 1–49%, EGFR T790M	7 mo.	11 mo.	8 mo.
3	Female	47	40 PY	Lung Squamous cell carcinoma	4-B	PD-L1 = 75–10%, KRAS G12D, STK11, CDC73	5 mo.	5 mo.	--

1PY = pack-year; mo. = months.

## Data Availability

Data is contained within the article or are available from the authors upon reasonable request.
